# Access at the doorstep: Supporting engagement with movement-based programmes

**DOI:** 10.1016/j.socscimed.2026.119479

**Published:** 2026-06-04

**Authors:** Emily Tupper, Tessa Pollard, Sarah Atkinson, Cassandra Phoenix

**Affiliations:** a Department of Sport and Exercise Sciences, Durham University, Green Lane, Durham, DH1 3LA, UK; b Institute for Medical Humanities, Durham University, Confluence Building, South Road, Durham, DH1 3LE, UK; c Department of Anthropology, Durham University, Dawson Building, South Road, Durham, DH1 3LE, UK; d Department of Geography, Durham University, Lower Mountjoy, Durham, DH1 3LE, UK

**Keywords:** Physical activity, Movement programmes, Cycling, Walking, Care, Ethnography

## Abstract

Voluntary, Community and Social Enterprise (VCSE) programmes that create opportunities to move outdoors via a volunteer-led activity offer a novel context through which to understand issues of access, participation and (re) engagement in structured physical activity or movement-based programmes. Through ethnography, this paper offers an account of the moments leading up to walking or experiencing a bike ride together in two different VCSE programmes. We focus on what is involved within and beyond the volunteer-beneficiary encounter in these programmes, and the different configurations of care that made participation and continued re-engagement in movement successful. We find that these configurations of care shifted moments of access, bringing to the surface the transitions, technologies, temporalities, and touch required of the volunteer-beneficiary encounter. Our findings demonstrate the importance of research exploring the “edges” of structured, movement-based activities, by showing what these moments reveal about participation, engagement, access, and care. We conclude by proposing that an aesthetic mode of enquiry within qualitative research could help inform the design, delivery, and evaluation of movement-based programmes or interventions.

## 1 Introduction: understanding access: bodies, environments, and thresholds

For those largely confined to their home, or living in care homes, physical activity (PA) and being outdoors are greatly valued for health and other benefits (Liljegren et al., 2024; Weening-Dijksterhuis et al., 2011). This has led to an interest in how PA or movement-based programmes help with varied health issues including those associated with loneliness and social isolation (Franke et al., 2021). However, the transition from home into the outdoors and movement can be challenging, and to date the processes involved have received little empirical attention. This paper uses ethnography to zoom in to the unfolding moments involved in such transitions, shedding light on what makes participation and re-engagement in movement-based programmes possible.

In describing these processes, this paper speaks to broader concerns regarding access to PA programmes for people experiencing limited mobility (Ball and Haegele, 2024; Brittain et al., 2020). However, rather than focusing on barriers and challenges to access, we follow Carroll et al. (2021) and Feely (2016) in their creative and experimental approach which they describe as discovering “more and more context-dependent capacities or things a body can do” (Feely, 2016:871). Our ethnographic focus allows us to do this via a novel context; movement-based Voluntary, Community and Social Enterprise (VCSE) led programmes which shifted the point of access to the boundary of the home; a walk or bike ride that begins at the doorstep. Focusing specifically on the unfolding moments of access across the threshold of the doorstep, this research offers new insights into what access and (re)engagement can look and feel like in practice and in place.

In this paper, we present detailed accounts of two different “moments” of access to show how the *volunteer-beneficiary encounter* is a powerful event because it has the potential to open up or shift the moments that lead up to participation in the activity. While these encounters are always specific to their local and organizational contexts, we show how focusing on the moments - and indeed micro-movements - immediately prior to (in this instance) a walk or bike ride, raises broader questions about care and access. Such insights hold great significance for structured movement-based contexts, which research has shown have varying degrees of success (Willinger et al., 2021). Data was collected via a range of methods; interviews (including go-along interviews), participant observation, photographic and visual data, and through an end of fieldwork discussion workshop. However, it is woven into a singular narrative voice here, a mode of storytelling typical of ethnographic writing.

Our focus on the “unfolding” of access as a creative endeavour draws conceptual inspiration from the notion of aesthetics, and specifically an *aesthetics of care*. Care aesthetics is ultimately a “provocation” to notice creative, artistic and social aspects to care practices that are often ignored or devalued (Thompson, 2022). It can operate in different ways and at different moments; as a concept, a framework, an approach and a practice (Maguire-Rosier et al., 2025:2). Although aesthetic concerns span many disciplines (Reynolds and Wiseman, 2018), several clusters of research and practice are relevant to this paper. For example, within the disability arts and disability justice movement, Cachia (2024) highlights curatorial practices that seek to create an “art of access, or access aesthetics”. This curatorial work challenges the idea of access as a singular event external to a physical space or building, instead centring it in a creative practice; a form of “translation” through a body that senses, and moves – and indeed encounters blockages and sticking points when attempting to move into different spaces. In this way, Cachia describes the mode of accessing art as *part of* the aesthetics; a process which is creative, embodied, and occurring in the immediate, unfolding moments. Meanwhile, the “translation” work this necessitates through the bodies of the audience (sensory expansion, touch, navigation and negotiation of micro-movements) can have value far beyond the immediate. Indeed, Cachia notes how such translation can offer the audience a lasting experience of ““being with” disability”.

In the health and social sciences, research exploring the relational engagement between the body and landscape has drawn on aesthetics to illustrate how this process is part of an “ecological aesthetic” that can have therapeutic or indeed “healing” effects (Hale et al., 2011). In the case of community gardening, this is because the body is engaged in a process of “ecological learning” which facilitates immediate, sensory engagement with the materials, rhythms and tempos of the garden, but in doing so, also affirms and expresses individual and cultural aesthetic values to do with health and the environment (ibid. 1855).

Meanwhile, writing on green and blue spaces within health geography also echoes how relational engagement with environments and materials matters for health, with the quality of spatio-temporal engagements in green spaces producing differential effects for people engaging in movement-based activity in these environments (Bell et al., 2014, Bell et al., 2015). Outdoor swimming practices involving different experiences of “immersion” have been similarly discussed as allowing a range of experiences from joy and healing alongside a sense of risk and fear for different people at different times (Foley, 2015). Together, this work affirms how the relationship between health and the moving body is made possible as part of wider ecologies, and in doing so, creates an arguably aesthetic account of health that is more than human. Research describing the communal therapeutic mobilities of group walking (Pollard et al., 2020) similarly has the effect of “opening up” how the moving body can effect health - often in ways that are unexpected and non-linear. This process occurs through somatic engagement in environments, which create opportunities to experience emergent, and sometimes collective, rhythmical effects that go beyond the individual body (ibid., Phoenix and Bell, 2019).

These varied and discipline spanning accounts of *how* engagement and access occurs between moving bodies and environments contribute to an aesthetic of health, and have implications for how we approach access in this paper. Specifically, an aesthetics approach within this context serves to broaden concerns around health and the moving body in ways that accommodate, methodologically, the emergent qualities of our immediate experiences, as well as more abstract and reflective accounts of why and how these experiences matter (Tainio, 2018). In these accounts, health involves engagements between bodies and environments in ways that are not necessarily pre-determined or even exclusively human. This creates a useful starting point for an ethnography of the moments of access described in this paper, to accommodate the unfolding nature of the volunteer-beneficiary encounter as it occurred across the threshold of the doorstep.

## 2 Methodology

The data presented in this paper comes from 12 months of ethnographic fieldwork within two VSCE-led programmes. The fieldwork took place predominantly in a city in the north of England from September 2018 to September 2019. This involved the lead author (ET) taking part in the programmes as a volunteer and as a researcher over this period.

### 2.1 The programmes

The programmes discussed in this paper are a paired walking buddy scheme (Move Mates: Home - Move Mates), and an innovative cycling initiative (Cycling Without Age: The Right To Wind In Your Hair - Cycling Without Age).

In Move Mates and Cycling Without Age, volunteers move with people who struggle to get out and about independently. Move Mates works on a referral basis for the beneficiary, and the focus is on their health and fitness, as their referral to the programme could be due to a lack of confidence getting out, fear of falling, or mobility problems. However, the volunteer is also expected to benefit from the experience, through simply “getting out”, regularly walking with someone, and perhaps building a friendship with someone they might otherwise not have met. Move Mates walks were often in close proximity to the home of the beneficiary and although adults of any age can request or be referred to Move Mates, many beneficiaries were older people.

Cycling Without Age, a global initiative which was locally organised by a women’s cycling group, involved volunteers helping older people living in residential care settings to get out and about on a bike. In Cycling Without Age, riding a bike becomes a shared experience; volunteer “pedallers” sit behind the older people on the bike – a seat which accommodates two passengers – and take them on a choice of various pre-planned and risk assessed routes. Although it involved physical effort from the volunteer, the electric assist feature on the bike meant that the rides were not intended to be concerned with improving the fitness of the volunteer, but more about enabling people of different generations and ages to experience movement, place, and fresh air together.

Whilst we discuss the volunteer-beneficiary encounter across the programmes in a general sense, it is important to note some key differences. Move Mates had the eventual goal of independent movement and health improvement; it aimed to “build confidence” in the beneficiary in a way that Cycling Without Age did not. Instead, Cycling Without Age was more about assembling “social participation” through the use of a specially designed bike (Lassen and Moreira, 2020). However, in both the programmes, we attend to the moments leading up to the walk or the bike ride, to unpack what is involved in taking part in structured movement-based activities for people experiencing ill-health and/or issues with mobility. In the examples in this paper, both were associated with older age, although we note Move Mates was not a programme specifically for older people. We look beyond the intended scope of the programmes, to understand these moments and what is required for them to be successful. We focus particularly on care, and the skilled care that was required, but which often fell outside of the scope of the programmes.

### 2.2 Data collection

On average,ET took part in 2-3 movement volunteering activities per week throughout the 12-month fieldwork period. These activities ranged from 1 to 3 h each. As well as participant observation, traditional and go-along interviewing methods, ET and participants used photographs taken as part of the activities as prompts for discussion in a group workshop at the end of the fieldwork period.

There were 24 participants in total across the two programmes; this included both volunteers, co-ordinators and beneficiaries (see Table 1 below). Move Mates was less established at the time of fieldwork hence the lower participant numbers. The data presented in this paper involves three beneficiaries, all of whom were older females (age 80+). Out of the 12 beneficiaries in the study, only one was male, and all were White British.

Ethnographic research aims to understand social phenomena “from the inside out,” embedding the researcher within relations, environments and cultures through long term, immersive participant observation. Ethnography has evolved from its beginnings in anthropology where the aim was to create holistic descriptions of specific groups of people (Nader, 2011). Instead, it can now be thought of as a “theory of description” (ibid.), taking place not just in bounded fieldsites but between places, in “non-places” (Gottschalk and Salvaggio, 2015) in hybrid settings, and, as was the case here, on the move. Moving bodies were central to the generation of the research data, and throughout the fieldwork, ET took extensive, descriptive, fieldnotes on how bodies moved within the programmes. These were usually written up as field-notes following the event, however some data was gathered “in situ” through mobile interviewing methods with participants.

### 2.3 Analysis

The process of analysis for this paper was heavily formed by the anthropological practice of reading and writing ethnography, which has its own relationship to the aesthetic (Hjorth and Sharp, 2014). In terms of the ethnographic text itself, it holds a particular set of stylistic and literary devices, for example the different ways of narrating the immediate and establishing authorship and authority through both presences and absences (Blasco and Wardle, 2019). For example, for this research we note as relevant to analysis here the *presence* of previous experience that the lead author had of sport and exercise contexts, as well as the *absence* of experiences of disability or care work. Although stylistic and literary, ethnography is different to poetry and novels in that the ethnographer is answerable for their ethnography as true knowledge (ibid. 201).

Analysis began with a close reading of fieldnotes and interview transcripts. Data was organised by the lead author as part of an iterative, sense-making process. This involved open-coding, whereby the lead author made descriptive notes on each data entry. This revealed a surprising amount of fieldnotes outside of the main movement activity. The first author coded this as “getting to the front door”, an “emic” code (Markee, 2013) which was brought into wider author discussions. Here, we discussed and theorised this as a significant embodied shift, thus initiating our “etic” engagement (ibid.) which allowed us to situate this code within wider interdisciplinary literature on care and access. Entry points offered by work on care practices (Mol et al., 2010) and care aesthetics (Thompson, 2022) helped us to articulate the different elements that work in synchrony to make access a situated, collective, and deeply embodied accomplishment. Through a close reading of Thompson’s work care aesthetics (2022) and alongside a workshop developed on this theme, we identified four live elements that unfolded in the volunteer-beneficiary encounter; transitions, technologies, temporalities, and touch. Through our analysis and discussion, we seek to destabilise the notion that moving more is always a “good thing” for health, because this discourse creates normative and ableist assumptions (Smith et al., 2021). We therefore build on disability and arts-based understandings of “access”, to take this work into new interdisciplinary spaces, alongside a description of care that is “ambivalent” in how it makes claims around increasing or optimising the movement of the body (Mol et al., 2010:11-14).

### 2.4 Ethics

The research for this project took place following departmental ethical approval in line with UKRI requirements. Participants were recruited via a consent form and information sheet, and received information regarding the purposes and storage of the research data. For anonymity purposes, all names have been changed.

Occupying the hybrid position of researcher and volunteer was useful for gaining an embodied insight into the programmes, and was an effective way to collect data within a novel and constantly evolving activity. However, researching as a volunteer meant being positioned within the values and objectives of the VCSE organisations. ET notes becoming actively involved in the development of the programmes through feedback, discussions, and volunteer meetings. Although this is an inevitable part of ethnographic methodology it required regular reflective practice on how the “slippage” between different roles i.e. volunteer and ethnographer influences the ethnography.

## 3 Findings: Creating movement possibilities in Cycling Without Age and Move Mates

### 3.1 Adjustments and manoeuvres

*Freya tells me to look out the window to see where she keeps her walker and her wheelchair. They are tucked away behind a bench in the shared courtyard space, under a shelter. Len and I go down and he shows me how he gets them out and sets them up. He lifts up the bench one side at a time, swivelling it round on one of the back legs so it takes most of the weight. He does it with the movements of someone who has done it many times before. He is only a few years younger than Freya, he tells me (he turns 80 next year), but he helps her out a lot. He sets up the walker and shows me how the brakes work. We walk it to the bottom of the steps and he puts it in position. Then it’s time for Freya to come down*.

*We go upstairs. Using the arms of the chair, Freya pushes herself to stand and they show me how she manoeuvres about the flat with her trolley - into the bedroom so that the bathroom door can be opened for example. They have worked it out so that she always has something to hold on to. She positions the trolley at the top of the stairs and then uses both of the banisters to walk down the stairs. Len reminds her not to rush. He is standing below her on the stairs and I am behind, at the top, following her steps down. They feel steep. We all comment on how going downstairs is actually harder - I say I find the same thing with running because it hurts my knees and Len says its the same with hillwalking* – *its because you have less control. Freya asks me how she is doing and I tell her she’s doing great and to take her time. She reaches the bottom of the stairs and leans forward into space until her hands meet the railings that accompany the external stone steps to her flat. Here, she pauses, and carefully pulls on her gloves. She uses both hands to do this, letting go of the railings, and her body sways slightly as she keeps her balance. She descends the stone steps one at a time. Len adjusts the mobility frame into a slightly better position for her and she grabs hold of it. We set off together round the courtyard*. (Fieldnotes, Move Mates, January 2019)

In one of our first Move Mates walks, I felt myself becoming orientated to Freya’s embodied experience of getting up and getting out to go for a walk, and how care “worked” to make this happen. In this example, it required a careful choreography of spaces, materials, and bodies, as well as an attention to the shifting qualities of the present moment, including the weather and how Freya felt from moment to moment. In my notes I describe how the positioning of everything was crucial, and the movements this required; for example the tilt and swivel of the bench outside in order to retrieve the mobility frame, the position, the angle and proximity of the frame to the bottom of the external steps, all of which were demonstrated to me by her friend and carer, Len. Clearly, the event of the walk caused a stirring of activity, indeed, even in its anticipation, because Freya was already dressed in her hat, scarf, and coat, all ready to go out when I arrived. Through the weekly walks, I became “orientated” to Freya and her space, the practiced embodied movements which allow her to negotiate the space of the flat, the stairs down to the front door, and the outside space of the courtyard. Everything was planned and practiced, every little space between the body and the surrounding environment was calculated and tested – not just by Freya, but in collaboration with Len, who offered a second perspective, another “pair of eyes” – or more accurately – a source of embodied knowledge. He would often use his own body to test out the spaces between things, and to demonstrate to Freya how to do each movement. Having known and cared for Freya for a long time, he knew the potentialities and limits of her movements better than I did. In the lead up to an operation on her arm for example, he had thought ahead about how she might get up and down the stairs, distributing her weight and using her legs more so that she could still get up and down.

It is important to acknowledge that Len and Freya were able to get out and about just the two of them – but what makes our walks a little different is the performative element. Whereas Len provided much of the tactile support (this is not something Move Mates are trained to do – physical support beyond walking arm in arm is outside the scope of the programme), Freya’s regular question; “how am I doing?” positioned me more as an outsider, or audience where I could feed back on the “performance”. Indeed, Freya used the word herself during my second visit, when she asked Len if he was also coming out to watch her “perform”. She was aware of her moving body being the main event, and actively positioned us as her engaged audience. Her walks with Len during the rest of the week gave them time to experiment with what worked and what didn’t, and when we were catching up about what each other had been up to during the week, their stories often centred around the processes and qualities of movement – not just where they had gone but how they had managed to get there.

Although the walks offered a challenge for Freya, which met her desire to “get better and better” as she described it, I found that in our pairing dynamic she was the expert from whom I was constantly learning. Indeed, we were less of a pairing and more of a triangular dynamic as Len also collaborated in this process, offering up demonstrations and instructions to both myself and Freya. Len was keen though, to foreground the Move Mates pairing of myself and Freya, and to take on as much of a background role as possible. This happened in a very literal way when Freya and I took part in some filming for a brief news bulletin about Move Mates. Len found much amusement in making a cameo appearance in the background, likening himself to the director Alfred Hitchcock and the way he cast himself as an extra in his own films.

This learning process of tuning in to how Freya moved was ongoing as her health continually fluctuated over our year of walking together. Small changes in weather, mood, or physical health that day had a huge bearing on the walks, which eventually became visits as her health deteriorated. Both myself and to a much greater degree - Len - were drawn into what has been termed “access intimacies”, which refers to the sense of safety felt by a disabled person when someone just “gets” their access needs (Mingus, 2011). It was these access intimacies between Len and Freya which meant Freya was able to continue to live in her home, despite the significant challenges of the stairs, the uneven paving externally, and the lack of designated storage space for her mobility frame.

Like the Move Mates walks, the Cycling Without Age rides cause a stirring of activity in their anticipation. “Getting out” becomes an event which is both planned but also constantly in motion. This excerpt comes from a few months in to the programme, where I now have a few “regulars” on the bike.

### 3.2 “Getting out”

*When we arrive at the care home there are loads of cars parked in the driveway, blocking our usual spot for parking Trixie (the electric trishaw bike). It means that the ramp usually used by the residents is blocked and I can’t bring the bike as close as I would like. I park Trixie and get the blankets out and the footrest off, to make it as accessible as possible and to minimise disruption in the transition on to the bike. Wendy (another volunteer pedaller) leans her bike against the pillars outside and we head in. The receptionist recognises us and calls for Ben, the care home wellbeing coordinator, on the radio. She says they have got a band on at the moment and we are to head on in because people might be busy. We go inside to the main atrium area - there is indeed a big band playing and residents are sitting around listening and socialising. We come across Mary who is dressed in her coat and scarf and bopping along to the music as she walks with her stick. She sees us and smiles and waves. This is the first time I have been further into the building to pick the passengers up for their rides. We then pick up Janice, who shuffles out her room with her walker. We edge slowly along the corridor. I try not to walk too far ahead of Janice, as I don’t want her to feel as if she is slow or holding us back. Mary says to me, oh I do love it when Ben comes to the door and says its time to go out! I say she’s not wearing two scarves this week like she normally does and she replies; no, its getting warmer isn’t it!*

*Once outside, they descend the other ramp this time as the usual one is blocked. It is a squeeze getting past all the cars, particularly with Janice’s frame. Janice goes in first as usual, sitting on the left of the bike (from my perspective behind her as the pedaller). I am holding the bike steady. I notice that her movements don’t appear any faster but Janice goes about getting on to the bike with a kind of familiarity and confidence now - with each ride her body must have worked out the spaces between things and how she must move into them, reaching out behind her for the side of the bike, backing in to the space where the footrest goes, sitting back on the seat, and shuffling along to her left to create space for Mary. She always says in a jokingly helpful way that she doesn’t mind her putting her hand on her knee if she likes, as Mary slowly backs into the seat next to her, rolling her eyes. She always remembers to shift her feet to the side for the footrest coming on, and reminds Mary to do the same. Ben and I tuck them in with the two blankets. He wonders out loud about getting the wheelchair for coming back in, for Janice. We leave her frame where it is, but Mary takes her stick with her as usual, holding it between her legs*.

*The act of “getting out” is a complex one here which requires co-ordination and effort both on the part of the care home and the volunteer co-ordinator in the lead up to the event; the consent forms, the risk assessment and “recce” rides, not to mention the training up of volunteers to ride the bike. After the advance co-ordination is the choreographing of the event itself, making sure the residents are ready to go out and that all the relevant mobility aids are in place. Much of this comes from the care home* – *the pedallers are not trained to physically manoeuvre or “transfer” the passengers (e.g. helping them get up) and as a result I tend to be an onlooker in this event, my only role being to steady the bike, applying pressure so that it doesn’t tip over when someone gets on or off. Then there is the transition onto the bike* – *the moment whereby time seems to slow down and the passengers work through the learned, habitual movements, with determination and resolve* – *not passive recipients of care but co-constitutions of the movement experience. Obviously once the passengers are on, seatbelts fastened, footrest in, blankets on, I am much more in control of the event; after all the shuffling and manoeuvring its always a lovely feeling “releasing” the bike, putting the throttle on a little, and moving out of the front drive. I do feel (as many of the passengers describe the rides) that it is their freedom, that they relish and treasure it, and I imagine what it must feel like to have been inside all day and then glide off on a bike into the sunshine* (Fieldnotes, Cycling Without Age, February 2019).

Although in residential care there are varying levels of independent living, moving into these care settings and the “institutionalisation” involved has been described as a form of “social death” (Taylor, 2010:36). Although the residential care setting in this paper offered a “wellbeing programme”, opportunities to “get out” and experience movement in fresh air were rare or non-existent for many residents, and it was the spontaneously sociable and sensory aspect of being outdoors on the bike that they particularly enjoyed. One resident, for example, described being “among people” and being able to “see the sky” as elements she took pleasure in. The being in - and moving through - outdoor, public spaces, is therefore an important part of care; facilitating a mobile way of engaging with places that hold meaning and pleasure for the person.

Like in Move Mates, movement was planned, anticipated, even feared – and this was felt in the body. Getting to the front door (and on to the bike in Cycling Without Age) required momentous effort, planning, and practice. The effects of movement were also felt in the body after the main event – this was talked about by beneficiaries of the Cycling Without Age programme as a feeling that “lasted” – suggesting that movement stays in the body internally somehow, forming waves or ripples which can be experienced for a long time afterwards. These descriptions suggest bodies are always in motion, just at different scales, intentions, and proximities to our awareness.

The practiced, repeated movements of the body as they shift towards the walk or bike ride in these contexts showed that anticipation is an important part of the rhythm of care. Rhythm involved a kind of dance between bodies as weight shifted and bodies responded to touch and movement. At a researcher-led workshop held at the end of the field-work period, volunteers identified “awareness of others movement” as a distinct way of moving within the programmes. This awareness did not just involve bodies; part of the rhythms of care in Cycling Without Age for example involved the electric-assist element of the bike, which softened the experience of one person moving another. This dynamic of being moved by the volunteer was joked about between “pedaller” and “passenger”, particularly when going up hills.

### 3.3 Jarring moments

There were, on occasion, jarring moments that further brought to the surface the co-created moments of moving together, and what could be at stake in these shifts into the bike ride or walk. For example, when becoming “stuck” in transitions (on and off the bike, or for Freya, the inside/outside boundary of her doorstep, which she found particularly challenging), the fragilities and vulnerabilities of moving together in the volunteer-beneficiary pairing was felt, as the volunteer improvised and requested assistance from other carers. The possibility of falling or becoming “stuck” was constantly present in these transitions. The volunteer-beneficiary dynamic then to some extent pre-determined the depth of access intimacies (Mingus, 2011) as they emerged between bodies in motion. The light touch of walking arm in arm in a short Move Mates walk (which was the extent of physical assistance volunteers were trained to offer outside of a first aid situation) involved attunement to rhythm, pace, and terrain, but it did not involve the extent of knowledge required of full-time or professional carers.

I learned of another jarring moment felt by a Cycling Without Age volunteer pedaller in an interview. Recalling her first experience of a Cycling Without Age ride, she cringed at riding the bike too fast, which she thought was a response to her feeling nervous. She said she soon realised that’s “not what the rides were about” – the passengers weren’t interested in getting from A to B quickly (which she said was her usual way of getting about on a bike around the city). Pedallers described a shift in how they cycled, which involved “tuning in” to bumps and textures of the road and cycleways. This attention and negotiation of pace and texture had an improvised quality, and was a skill that was constantly being developed throughout the rides.

There was also some confusion about what should happen with mobility aids when the passenger was on the bike. Most of the time, it was not possible or practical to take larger aids on the bike. Because of this, the rides were fairly short (approximately 30 min), with them all starting and finishing at the care homes. But Mary, mentioned in the earlier excerpt, always liked to take her walking stick with her, tucking it between her legs once she was seated. I remember the discomfort I felt when one of the care home staff facilitating the transition onto the bike reached forwards to take it from her once she was seated with the reasoning that she wouldn’t “need” it. Mary didn’t object to this but there was something that felt wrong about it. Care then, can also involve a care of things, aids, and objects that are required for access and transitions to feel as familiar and comfortable as possible.

The importance of attending to people’s belongings has also been described in ethnographic work within a wheelchair test centre (Winance, 2010). Winance describes the adaptations and empirical tinkering (Mol, 2006; Pols, 2004) involved in the process of choosing a wheelchair for a patient. She observes that the provider of the service in the hospital, Benoit, notices how “Mrs S” likes to have her bag next to her on the chair and asks if this is important to her – to which she confirms it is. In reflecting on his role in the test centre, he says “Le mieux est l’ennemi du bien” – or “the perfect is the enemy of the good” and that whilst for Mrs S the narrower chair would be “perfect”, when taking into account her preference to be close to her belongings, this means providing a wider chair that would not fit her body so well. Winance reflects on what this tells us about “good care” – that it is not perfect, but a compromise; “the good is an arrangement of people and things that is a compromise, allowing a life together and allowing motion and emotion for all those involved in the collective” (ibid. 109).

### 3.4 Summary

Working outwards from the volunteer-beneficiary encounter, we have drawn attention to the access and the care that this encounter required. We show how moving bodies were co-created via interdependencies with environments, tools, technologies, and other bodies. Moving the body within a volunteering activity created possibilities for both volunteer and beneficiary to move in different, and valued, ways. Care, in these movement-based contexts, could involve moving at the same speed or rhythm, it sometimes required mirroring someone else’s movement, pacing someone, or perhaps consciously slowing down. In some cases – for example in the case of bad weather during a bike ride - it might involve moving faster. It required with-holding, releasing, and sharing energy in both intentional and unin-tentional ways, as well as caring for and with objects, aids, textures, and landscapes.

## 4 Discussion

The diverse and varied ways in which movement entwines with care in these contexts supports disability arts and activist-based understandings of access as intimate, aesthetic, and creative, and requires our methodologies to attend to it as such. However, this can pose a challenge when researching movement-based activities that have a structured element (i.e. a time, a place, and a stated purpose), as these activities often lend themselves to positivist research frameworks, for example ones that focus on participation and non-participation, and can lead to discussions around seemingly “fixed” barriers and enablers (Haynes and Loblay, 2024:5-6). Because of the novel volunteer-beneficiary encounters created by these VCSE-led programmes, we take the opportunity to shift our focus into what may be happening in these relational, and sometimes fragile, moments of access; what did these encounters open up, or shift? Why and how did they enable continued re-engagement in the programmes over the fieldwork period?

We find that whilst these volunteer-beneficiary encounters created important opportunities to move in a different way, to successfully transition into the walk or bike ride required a cluster or “configuration” of care practices, involving “tinkering”, and sometimes compromise (Winance, 2010; Mol et al., 2010; Pols, 2004). Care is therefore embodied, crafted, and sensory – making the case for an “aesthetics” of care (Thompson, 2022) operating in these contexts. In this ethnographic study we found care aesthetics resonated throughout the research process, in practice (getting to the front door) in analysis (to identify what elements made getting to the front door successful) and also, as we now present, to influence policy and practice by providing a set of considerations to guide accessibility initiatives in sport, physical activity and health contexts. For example, this could include social prescribing into “green” activities such as outdoor walking and gardening groups which have seen limited success due to lack of considerations of the nuances of access in these contexts (Pollard et al., 2025).

### 4.1 Transitions

As mentioned previously, it is significant that volunteers were not trained to physically support and move people, and this set up boundaries in the volunteer-beneficiary relationship which prioritised leisure-oriented experiences of being and moving together in outdoor contexts. In these contexts, the transition into moving outside was grounded in a series of movements which disrupted existing ways of being in the body, and existing patterns of caring and being cared for. There were moments in transitions when people did become stuck, and in these moments, preexisting care arrangements were important. It was important that the care home staff were available to support the transition on to the bike, for example, and Freya relied on Len to physically help her get to the front door, and set up her mobility walker. Although the volunteer/ beneficiary relationship set a context for moving the body that was intended to be enjoyable, the leisure-based activity of moving outdoors was scaffolded on an aesthetics of care that already existed in some form, but which was constantly being tinkered with and negotiated in order to make access into the activity possible. The combined physical support and conversation between the cycling programme beneficiaries and care home staff to support the transition on to the bike, for example, and the careful choreography of Len and Freya’s movements through Freya’s flat: This was an integral part of access into the programmes.

However, it is important to note that a lot can be at stake in those transitions, such as bodily autonomy, personhood and feelings of fear or discomfort when mobility aids or people’s belongings are not treated as important. The programmes then, did not simply set up the transition into movement, they also set up shifts between different configurations of care; experiences of moving together in a walk or cycle ride build on existing access intimacies (Mingus, 2011) including skilled care work, which operated in synchronicity to afford experiences of leisure and movement in the volunteer-beneficiary encounter.

### 4.2 Technologies and tools

Technologies were an important element of the programmes, but their use depended on relations of care. For example, although the electric trishaw enabled the main – and novel - event of the Cycling Without Age ride, it wasn’t the starting point - care was the starting point. Even the use of the electric trishaw in the first place was dependent on care of the bike by a cycle shop who serviced and stored it.

Bikes hold many possibilities to configure patterns of care and ways of relating to one another (see Lassen and Moreira (2020) and Hammer (2015)). On the other hand, mobility aids were simultaneously relied on and resented, reliable and unwieldy. Technologies can be both in sync and out of sync with how people want to move their bodies, and good care in these contexts meant tinkering, adaptation, and compromise (Winance, 2010; Mol, 2006; Pols, 2004). Also important were configurations of domestic spaces and the care within and of them, in setting up the possibility to get to the front door in the first place.

Objects and technologies associated with care can be experienced differently by different people – as Thompson illustrates in the example of being asked by a friend and colleague from the Democratic Republic of Congo why children in the UK are pushed about in wheelbarrows, facing away from their parents (Thompson, 2022:1-2). Arguing that they are in fact pushchairs does not answer the question about how we care and move together with infants, nor does it help us understand the relations that are produced and reproduced in this process (ibid.). In an aesthetics of care, it is not always possible to discern where body and material stops and starts; where a “physical environment ends, where health policy acts on a situation or is changed by it, and where people experience care because of or in spite of the multiple elements of its spatial arrangements” (Thompson, 2022:134). Tracking this experience in these volunteering contexts reveals not only the importance of creativity in designing technologies that aim to create pleasurable experiences of movement, but the importance of creativity in embedding technologies in relationships, practices, and places.

### 4.3 Temporality and scale

The programmes co-created moments which offered a different experience of time, or “time out” for both volunteers and beneficiaries. As a volunteer in the programmes, I felt as if time slowed down through the mobile encounters of the bike rides and walks. I found this tuning in and change of pace and scale could often feel therapeutic, and many of the volunteers also described the pleasures of slowing down and “noticing” the environment more than they would normally. However, for the beneficiaries, transitions were challenging, and required skill, resolve, and practice. Freya often voiced feelings of fear in the transition out of the front door, and there was a skill in moving through these moments that meant they weren’t one-directional but “co-created” (Thompson, 2022:123). Although we have used the term “beneficiary” for ease and to follow the language used by the VCSE programmes, there was considerable work involved in performing the “beneficiary” role.

These co-created moments meant care time was unpredictable and did not line up with clock-based time (Thompson, 2022:136). So how did this play out in volunteering contexts where sensitivity to rhythm and pace was so important? In the VCSE contexts, the organisations were particularly concerned with protecting and drawing boundaries around the volunteering encounter. This was both in the interests of volunteer recruitment and retention, as well as strategic focus on charitable aims and objectives. Because of this, the programmes set out certain time limits which had a protective effect on – or simply measured -volunteer time. This was intentional, to make the volunteering easy and appealing for the volunteer – something which seemed to work, given the successful volunteer recruitment process that I observed during the field-work period. However, protecting the volunteer encounter and thus the boundaries of the programme scope also meant a heightened need for well-funded and skilled care work that could continuously attend to the access needs of the encounter.

The valued “time out” therefore held a range of meanings for people, and scale and temporality was experienced in different ways throughout the programmes. On the other hand, even the possibility for there being a “time out” in the first place, was contingent on everyday care work from friends, family, and care workers across different settings. These findings may provide useful insights for public health initiatives that aim to “scale up” physical activity or movement-based programmes (Beedie et al., 2014). Whilst it may seem straightforward to grow a programme that seems to be working, it can be difficult to disentangle the different configurations of care that make that programme appear successful.

### 4.4 Touch

Manning describes touch as the meeting of two bodies, the invention of worlds between people (Manning, 2007:60). We follow this understanding of touch; as a generative and creative encounter. Tuning in to moments and uses of touch in our analysis of the volunteer-beneficiary encounter reminds us that our engagement with the world is “bodily”, and that there is always translation at work when we try to write up or describe experiences of access, particularly (in this case) using ethnography. Nevertheless, we notice the diversity of different bodies and environments that are “in touch” in these mobile encounters.

Moments of touch in the volunteer-beneficiary encounter revealed the considered and embodied preparations that were involved in setting up the transition into the walk or bike ride; whether that was the way the hand reached backwards to find the edges of the seat on the bike, the touch of an arm, or the daily tilt and swivel of a bench in order to retrieve a mobility aid. In setting up possibilities to move together, the VCSE programmes mirrored curatorial work which seeks to create an “art of access” and offering experiences of “being with” disability (Cachia, 2024). However, without acknowledging the aesthetics of this work in these everyday spaces, we lose touch with what might be possible in creating good access for structured movement-based programmes.

## 5 Conclusion

In this paper, we use ethnography to zoom in to the moments leading up to the walk or bike ride in two different movement-based programmes which co-ordinated volunteer-beneficiary encounters. We find that access involved skilled care which often fell outside of the programme scope. However, by focusing on moments leading up to the walk or bike ride, we make the case for bringing these in from the peripheries in ways akin to recent curatorial practices that make access the subject matter (Cachia, 2024:8) and call on qualitative work in sport and physical activity contexts to explore what is going on experientially in these moments. Our theoretical work here brings together arts-based understandings of access to the fields of physical activity and health to open up a more expansive and inclusive account of how physical activity relates to health and could work towards addressing concerns around accessibility and ableism in sporting and physical activity contexts.

Applying the concept of aesthetics is both original and valuable in terms of articulating the relational aspects of care, access, and movement that came to the fore in the volunteer-beneficiary encounter. We see this as contributing to an existing aesthetic account of health and the moving body that acknowledges the emergent effects of the moving body and challenges accounts of health that are overly deterministic. We suggest that an aesthetics of care as set out by Thompson (2022) could become a central concern for the successful design and delivery of movement-based programmes, and offer four elements that emerged from our ethnography; transitions, technologies, temporalities and touch. However, we note our ambivalence of the links between moving bodies and health outcomes in these contexts – what matters here is creating an aesthetics of access that attends to these elements as a starting point. We also note our study is limited in scale and diversity of participants in terms of age, gender, and ethnicity. Future work is needed to explore how multiple social identities may experience these moments of access and to explore how our proposed care aesthetics framework may shift and change in dialogue with these experiences.

This paper also develops and expands existing theoretical work that acknowledges the entanglements of the moving body and care. We bring together work on care practices and care aesthetics (Mol et al., 2010; Thompson, 2022) with work that describes either implicitly or explicitly the aesthetics of moving the body in and for health to show the varied ways in which moving bodies could be connected to health. By opening up an aesthetic mode of enquiry, we have generated a flexible analytical approach that attends to diverse experiences of engagement in movement-based activity. We also hope this acts as a stimulus for similar work in the future that explores how aesthetic concerns could play into the design, delivery, and evaluation of movement-based or physical activity programmes or interventions. For example, this approach may be useful for understanding the efficacy of referral pathways found in social prescribing, or other interventions which anticipate beneficial outcomes from movement-based activity outside of a clinical setting. Further interdisciplinary work is also needed to understand whether and how specific movement practices *e.g*. walking or cycling, could be brought into encounter with their own histories and aesthetic forms.

## Figures and Tables

**Table 1:** Participants.

	Total	Volunteers and co-ordinators	Beneficiaries
Cycling Without Age	17	7	10
Move Mates	7	5	2

## Data Availability

The data that has been used is confidential.
